# Carotid artery calcifications on panoramic radiographs are associated with vascular disease severity on carotid ultrasound

**DOI:** 10.1093/dmfr/twaf061

**Published:** 2025-09-12

**Authors:** Astrid Karlsson, Nils Gustafsson, Per Wester, Liene Zamure-Damberga, Eva Levring Jäghagen

**Affiliations:** Oral and Maxillofacial Radiology, Department of Odontology, Umeå University, SE-90187 Umeå, Sweden; Department of Oral and Maxillofacial Radiology, Postgraduate Dental Education Center, SE-70115 Örebro, Sweden; Oral and Maxillofacial Radiology, Department of Odontology, Umeå University, SE-90187 Umeå, Sweden; Department of Public Health and Clinical Medicine, Umeå University, SE-90187 Umeå, Sweden; Department of Clinical Sciences, Karolinska Institutet, Danderyd Hospital, SE-18288 Stockholm, Sweden; Oral and Maxillofacial Radiology, Department of Odontology, Umeå University, SE-90187 Umeå, Sweden; Department of Conservative Dentistry and Oral Health, Faculty of Dentistry, Riga Stradins University, Riga LV-1007, Latvia; Oral and Maxillofacial Radiology, Department of Odontology, Umeå University, SE-90187 Umeå, Sweden

**Keywords:** radiography, panoramic, ultrasonography, carotid arteries, accuracy

## Abstract

**Objectives:**

The aim of this study was to investigate whether any feature of carotid artery calcification (CAC) detected on panoramic radiographs (PRs) is associated with more severe signs of cardiovascular disease (CVD), as assessed by carotid ultrasound (CUS) including multi-view assessment of carotid intima media thickness (cIMT).

**Methods:**

The present investigation was a retrospective sub-study of the randomized controlled trial visualization of asymptomatic atherosclerotic disease for optimum cardiovascular prevention (VIPVIZA), which included 60-, 50-, and 40-year-old inhabitants of Västerbotten County, each of whom underwent CUS. The present sub-study included 135 participants who had undergone PR for odontological indications within 18 months before or after CUS examination. Findings of CAC on PR were compared with CUS findings of cIMT and carotid plaque. CAC features were categorized into 4 types: single, scattered, vessel width-defining, or vessel-outlining.

**Results:**

Compared to participants without CAC on PR, those with any CAC type on PR exhibited significantly more carotid plaque (80.9% vs 43.2%, *P* < .001) and a higher average cIMT score (0.83 vs 0.77 mm, *P* = .013) on CUS. The vessel-outlining CAC group exhibited the most pronounced cIMT and carotid plaque occurrence (*P* = .011).

**Conclusions:**

All CAC types detected on PR were associated with CVD on CUS, and vessel-outlining CAC indicated more severe CVD. By detecting CAC on PR, especially vessel-outlining CACs, dentists could contribute to the early identification of patients with asymptomatic CVD, and recommend that these patients seek medical attention for preventive treatment.

**Advances in knowledge:**

All types of CAC detected on PR—particularly the vessel-outlining type—are associated with carotid ultrasound findings, including carotid intima media thickness, indicating CVD and increased risk of stroke and myocardial infarction. Thus, dentists can identify patients at increased risk of cardiovascular events by detecting CAC  on PR, with higher diagnostic reliability in cases with vessel-outlining calcification.

## Introduction

Cardiovascular disease (CVD) is the leading cause of death and disability worldwide, substantially contributing to global disease burden and healthcare costs.^1^ The main underlying cause of CVD is atherosclerosis, a chronic inflammatory disease of the arterial walls, initiated by cholesterol-rich lipid accumulation and the corresponding inflammatory response, as well as accumulation of cellular debris and calcium, which can ultimately cause stenosis or thrombosis.^2^

The degree of atherosclerosis in the carotid arteries can be assessed by carotid ultrasound (CUS), a validated non-invasive procedure that provides measurements of plaque presence and carotid intima media thickness (cIMT). Both carotid plaque presence and increased cIMT are associated with increased risk of cardiovascular events, such as stroke and myocardial infarction.^3^ CUS-determined cIMT is a valuable measurement for quantifying subclinical vascular disease, and assessing whether a patient has an increased risk of future cardiovascular events.^4^ Atherosclerosis usually has a long asymptomatic phase, and over half of affected individuals are unaware of their atherosclerosis until it causes severe CVD events. Such events can be avoided if presymptomatic atherosclerosis is identified and preventive treatment initiated during an early stage.^5^

Carotid artery calcifications (CACs) are a sign of atherosclerosis, which can be incidentally detected by dentists on panoramic radiographs (PRs) obtained for odontological indications. These incidental findings provide an opportunity for early diagnosis and preventive treatment among patients who may otherwise be asymptomatic until they suffer a cardiovascular event. A PR depicts the teeth, jaws, and surrounding structures, often including the neck and carotid arteries, enabling the detection of calcifications as small as 1 mm^3^ in carotid plaques.^6^ Previous studies show that CACs found on PRs are associated with carotid plaques detected by CUS.^7^ One cross-sectional study revealed that 84% of the patients with significant carotid stenosis (≥50%) also exhibited CACs on PRs. Moreover, among 101 extirpated plaques, 100 (99%) were calcified.^7^

Compared to individuals with other types of CACs on PR, those exhibiting bilateral vessel-outlining CACs reportedly have higher cardiovascular risk, and a greater likelihood of major adverse cardiovascular events.^8^^,^^9^ However, in these studies, CUS was performed only in participants who showed CACs on PR. Another study also demonstrated that different features of CACs detected on PR are associated with the degree of CUS-detected plaque findings suggestive of CVD—with vessel-outlining CACs associated with plaque characteristics indicating more severe atherosclerosis on CUS.^10^ However, these previous studies have not included comparisons between different features of CAC on PR and CVD assessed by CUS measures of cIMT. There remains a need for studies including participants both with and without CACs on PRs, and examined using CUS, to investigate the associations between cIMT and different types of CACs detected on PR.

The VIsualiZation of asymptomatic Atherosclerotic disease for optimum cardiovascular prevention (VIPVIZA) study is a pragmatic randomized controlled trial nested in the Västerbotten Intervention Program (VIP), which includes healthy inhabitants of Västerbotten County enrolled during 2013-2016.^11^ The participants in VIPVIZA comprised 60-year-old individuals, and 40- or 50-year-old individuals having at least one established CVD risk factor. These subjects underwent a thorough medical examination, including CUS assessment of the cIMT and for the presence of plaque, at baseline and at 3- and 6-year follow-ups. The aim of the VIPVIZA study was to investigate whether personalized pictorial information about carotid artery status, including guidance regarding CVD prevention, would result in reduced cardiovascular risk compared to controls.^11^ At the 6-year follow-up, the participants underwent the third CUS, and were invited to participate in a dental sub-study.

Here we report this dental sub-study of the VIPVIZA study, in which we aimed to investigate whether any feature of CACs detected on PR was associated with more severe signs of CVD, as assessed by CUS, including findings of cIMT.

## Methods

### Ethical considerations

This dental sub-study was included as an amendment (2019-04691) of the VIPVIZA study (2011/445-31), and was approved by the Swedish Ethical Review Authority. All participants provided written informed consent for participation at inclusion in the VIPVIZA study, and for participation in the dental sub-study at the 6-year follow-up.

### Participants

**Figure 1.**
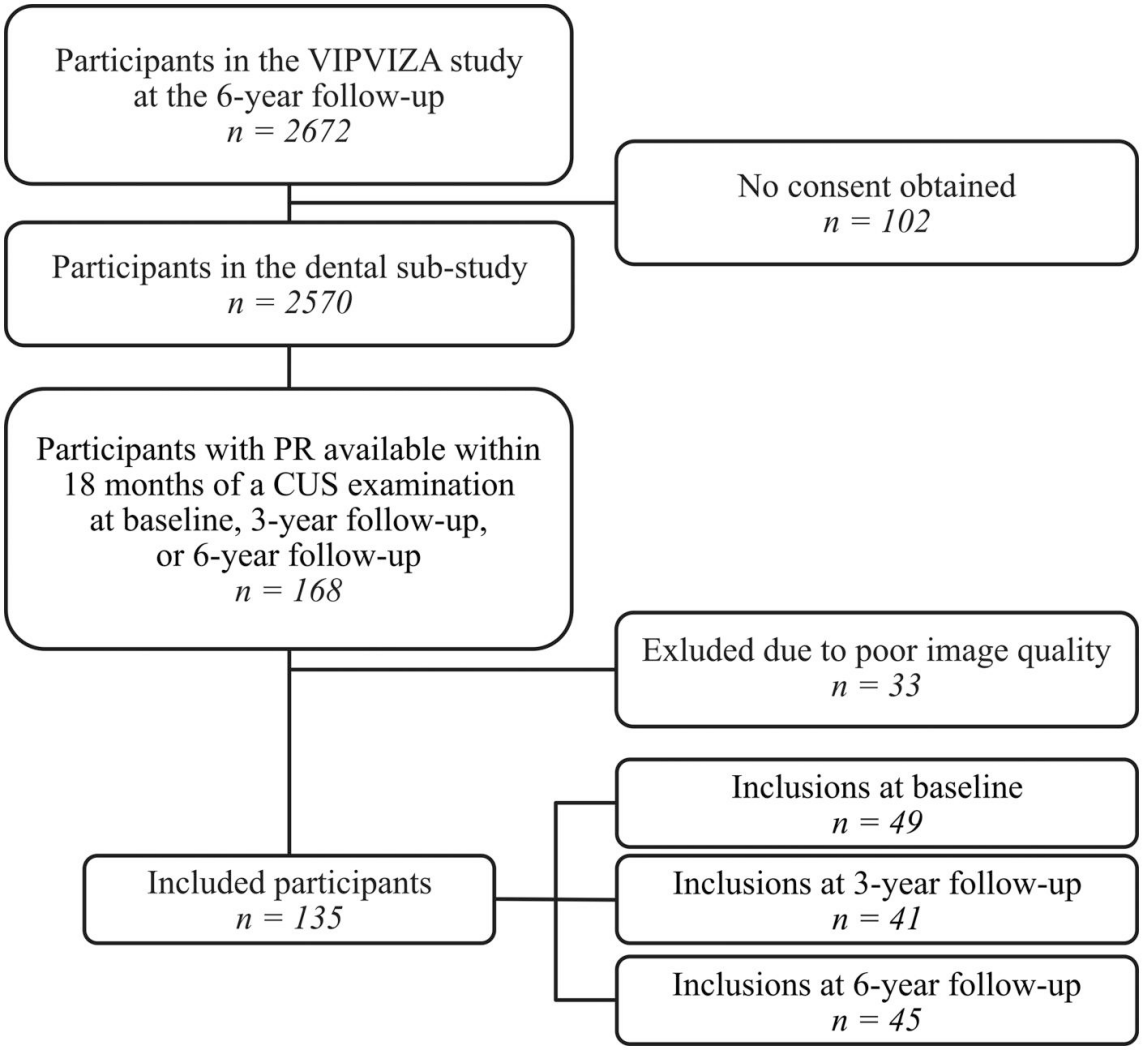
Flowchart of patient inclusion. PR = panoramic radiograph, CUS = carotid ultrasound.

At the 6-year follow-up, 2570 of 2672 eligible VIPVIZA participants agreed to participate in the cross-sectional and retrospective dental sub-study. For these participants, we assessed all dental records and collected all radiographs, including PRs. A total of 168 participants were found to have undergone panoramic radiography for odontological indications, at public or private dental clinics in Västerbotten County, within 18 months before or after baseline or the 3- or 6-year follow-up examinations (Figure 1).

### Ultrasound examination

In the VIPVIZA study, CUS examinations were performed at baseline and at the 3- and 6-year follow-ups by 6 calibrated sonographers with 4-20 years of experience performing CUS. The inter-sonographer reproducibility for plaques had a kappa value of 0.70, using Cohens kappa,^12^ which was established when the sonographers scanned-rescanned the same participants immediately after each other in separate rooms, blinded to each other’s results. Before initiation of the VIPVIZA study, another study using the same equipment evaluated the reliability of the cIMT measurements, and showed intra- and inter-sonographer agreement coefficients ranging from 0.91 to 0.95.^13^

**Figure 2.**
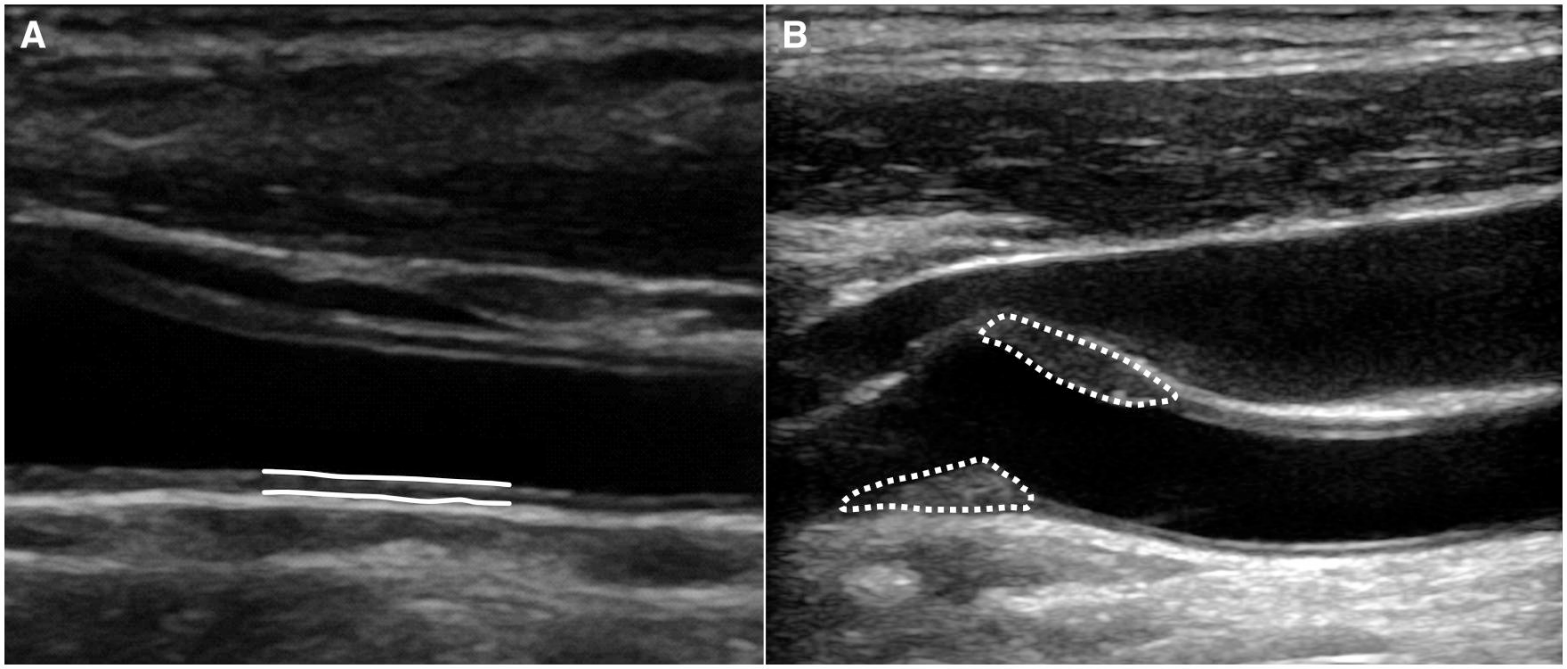
Carotid ultrasound examinations showing measures of carotid intima media thickness (cIMT) and presence of plaque (yes/no). The cIMT was automatically measured in a carotid intima media section with a length of approximately 1 cm. The cIMT was measured in millimetres, the maximum and mean cIMT of all projections (120, 150, 210, 240 degrees) was registered for each neck side, and the mean value of the highest maximum of both sides (right and left side) was registered for each participant. A plaque was registered as present when focal thickening of the cIMT was present, or if the IMT exceeded 1.5 mm, in accordance with the Mannheim consensus. (A) Solid lines outlining a measured section with normal cIMT. (B) Dotted lines indicate plaques.

The common carotid arteries on both sides of the neck were scanned at insonation angles of 120, 150, 210, and 240 degrees, yielding automatic measures of the cIMT in millimetres, and recording the maximum and mean cIMT of the projections of each neck side, and the mean value of the highest maximum of both sides (right and left side) was registered for each participant. Atherosclerotic plaque presence was recorded according to the Mannheim consensus, ie, when a focal increase of cIMT was detected, or if the cIMT exceeded 1.5 mm^11^ (Figure 2).

Two different types of portable CUS equipment were used—the Panasonic CardioHealth Station at baseline and the 3-year follow-up,^11^ and GE Vivid iq at the 6-year follow-up.^14^ This change was made due to an equipment upgrade and the evolution of technology over time during the study period. Calibration was conducted and measures were taken to ensure consistency.

### Panoramic radiographs

Each PR was exported from the patient’s dental record as an uncompressed TIFF file, and assessed by 2 experienced researchers and specialists in oral and maxillofacial radiology (ELJ and NG), who respectively had >30 and 10 years of experience in diagnosing CACs on PR. Both were blinded to information about the participants. They separately assessed the PRs in a dimly lit room, using high-resolution and high-quality displays with the ability to adjust the brightness, contrast, and magnification. In cases of disagreement regarding CAC presence, consensus was reached through discussion. The inter-observer agreement for CAC detection and categorization of CAC type were nearly perfect with kappa values of 0.950 (95% confidence interval [CI]: 0.922-0.987) and 0.897 (95% CI: 0.861-0.933), respectively.^15^

**Figure 3.**
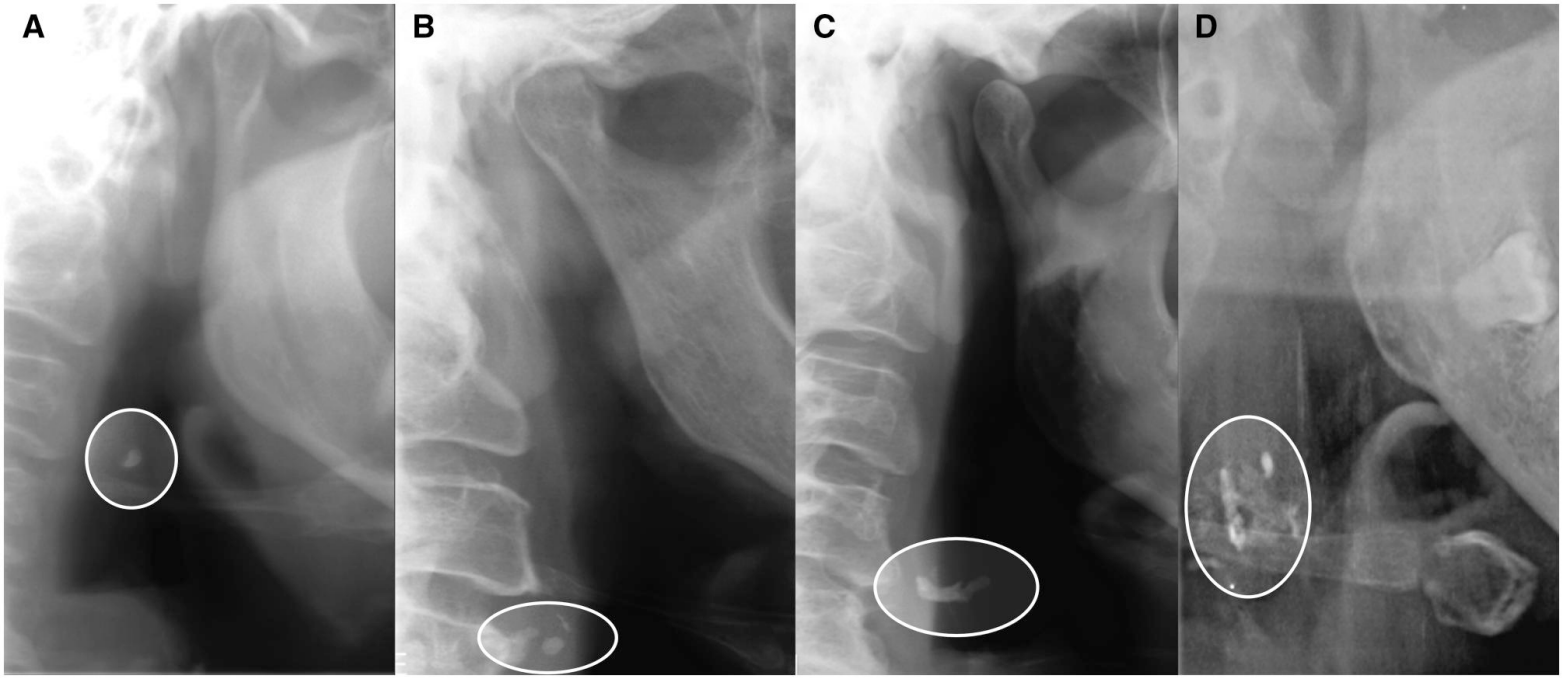
Carotid artery calcifications on panoramic radiographs. (A) Type 1, single calcification. (B) Type 2, two or more scattered calcifications. (C) Type 3, vessel width-defining calcification. (D) Type 4, vessel-outlining calcification.

The PRs were assessed for CACs on both the left and right side. Detected CACs were further stratified into 4 types based on features: type 1, single calcification not matching any other described type; type 2, two or more scattered calcifications not matching the description of type 3 or 4; type 3, vessel width-defining calcification, showing the vessel width but not extended to outlining the vessel; and type 4, vessel-outlining calcification, showing the vessel width and part of the height (Figure 3). For participants presenting with bilateral CACs of different types on each side of the neck, the highest category was used for data analysis.

### Statistical analysis

All statistical analyses were performed using SPSS Statistics 29.0 (IBM SPSS). All continuous data were assessed for normality using the Shapiro-Wilk test. Diagnostic accuracy was evaluated by calculating the sensitivity, specificity, positive predictive value (PPV), and negative predictive value (NPV). The inter-observers’ agreement was calculated using unweighted Cohen’s kappa (κ) for both 2 and 5 categories.^15^ Participants’ background characteristics are presented as mean values with standard deviation, minimum and maximum values, and percentages. Background characteristics were compared between participants with and without CACs on PR, and with and without plaques on CUS. Differences in categorical variables were analysed using the two-sided χ^2^ test. Differences in continuous variables were analysed by 2-tailed Student’s *t*-test if the data were normally distributed, or the Mann-Whitney U-test for non-normally distributed data. Moreover, for non-normally distributed data, the participants’ background characteristics were also presented as the median and interquartile range (IQR). Relationships between cIMT and different types of CAC were evaluated using either a 2-tailed Student’s *t*-test or Kruskal-Wallis test, depending on the number of samples and distribution. Relationships between plaque presence on CUS and CAC presence on PR were analysed using the 2-sided χ^2^ test. The Bonferroni correction was used for multiple comparisons. *P* values of <.05 were considered significant.

*Post-hoc* power analyses, using an alpha level of 0.05 and a target power of 0.80, were conducted for both the independent samples t-test and the Wilcoxon Mann-Whitney U-test. These analyses revealed the need for 50 participants in each group for comparison between no CAC versus any CAC, and 14 in each subgroup for comparison between vessel-outlining CAC versus no CAC. Similarly, when the effect sizes were calculated based on a previous study,^16^ the estimated required sample size ranged from 24 to 26 participants per group for comparison between CAC versus no CAC.^16^ All of these results suggest that our sample size is adequate.

## Results

### Participants

The present analysis included 135 of the 168 participants who had undergone panoramic radiography during the VIPVIZA study period at the 6-year follow-up. These 135 participants comprised 73 women (54%) and 62 men (46%) with a mean ± SD age of 60.7 ± 5.7 years (Table 1). The remaining 33 patients were excluded because the PRs exhibited overexposure or poor quality (*n *= 15), failed to depict the area of the carotid arteries (*n *= 17), or showed artefacts due to patient movement (*n *= 1) (Figure 1). When a participant had multiple PRs available, we selected the PR obtained closest in time to the CUS examination. Two included PRs were only interpretable on one side of the neck, because the PRs were segmented at exposure.

**Table 1.** Demographic differences between groups with and without carotid artery calcification (CAC) on panoramic radiographs (PRs), vessel-outlining CAC on PRs, and presence of plaque on carotid ultrasound (CUS).

		All included participants	No CAC	All CAC types	*P* value	Vessel-outlining CAC	***P* value** *	Plaque	No plaque	*P* value
		*n *= 135	*n *= 88	*n *= 47		*n *= 16		*n *= 76	*n *= 59	
Age, years	mean (SD)	60.7 (5.70)	59.6 (5.70)	62.7 (5.18)	**.002** ^a^	64.9 (2.78)	NA	61.5 (5.49)	59.7 (5.85)	.072^a^
	median (IQR)	–	–	–	NA	64.5 (63-68)	**<.001** ^b^	–	–	NA
	min-max	42-69	42-68	50-69	NA	60-69	NA	42-69	48-68	NA
Sex, female	*n* (%)	73 (54.1)	43 (48.9)	30 (63.8)	.096^c^	11 (68.8)	.143^c^	38 (50.0)	35 (59.3)	.281^c^
Months between PR and CUS	mean (SD)	8.71 (5.29)	8.47 (5.11)	9.17 (5.64)	.463^a^	10.13 (5.6)	NA	8.29 (5.58)	9.25 (4.89)	.295^a^
	median (IQR)	–	–	–	NA	10 (6.25-15.75)	.293^b^	–	–	NA
	min-max	0-18	0-18	1-18	NA	1-18	NA	0-18	0-18	NA

* Compared with no CAC.

a Student’s t-test, two tailed.

b Mann-Whitney U-test.

c Pearson’s chi-squared.

Abbreviation: NA = not applicable.

**Bolded *P*-values** are statistically significant (*P* <.05).

### Distribution of carotid artery calcifications

Among the 135 analysed PRs, 47 (34.8%) showed CAC, 16 (11.9%) of which were vessel-outlining CAC. Participants without CAC on the PR had a lower mean age (59.6 ± 5.7 years) compared to those with any CAC type on the PR (62.7 ± 5.18 years, *P* = .002). Furthermore, participants presenting with vessel-outlining CAC (type 4) exhibited an even higher mean age (64.9 ± 2.78 years; median 64.5 years, IQR 63-68) compared to those without CAC (*P* < .001). Participants with versus without CAC did not significantly differ in sex or months between the PR and CUS examinations (Table 1).

The participants were assigned a single value based on the highest CAC type observed on either side of the neck, including the 2 participants for whom only one side was interpretable. Table 2 presents the prevalence of different types of CAC detected on PRs, at the individual level and according to the side of neck. We found that 13 participants (9.6%) had type 1 CAC on one or both sides of the neck, 10 (7.4%) had type 2 on at least one side, 8 (5.9%) had type 3 on at least one side, and 16 (11.9%) had type 4 on at least one side (Table 2).

**Table 2.** Prevalence of different types of carotid artery calcification (CAC) detected on panoramic radiographs (PRs), according to side of neck and at the individual level.^a^

CAC types detected on PRs		
*Neck sides*	*n*	%
No CAC	202	75.4
Single calcification (type 1)	22	8.2
Scattered calcification (type 2)	11	4.1
Vessel width-defining calcification (type 3)	11	4.1
Vessel-outlining calcification (type 4)	22	8.2
Total	268	100
*Individual level (highest score on either neck side)*		
No CAC	88	65.2
Single calcification (type 1)	13	9.6
Scattered calcification (type 2)	10	7.4
Vessel width-defining calcification (type 3)	8	5.9
Vessel-outlining calcification (type 4)	16	11.9
Total	135	100

a Two participants were missing one side of the neck on PRs, due to segmentation.

### Carotid ultrasound findings of cIMT

**Figure 4.**
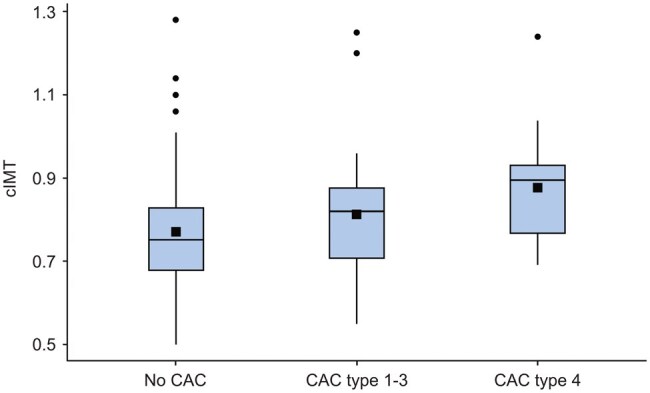
Carotid intima media thickness (cIMT) from carotid ultrasound examinations, in relation to no carotid artery calcification (CAC) and different types of CACs detected on panoramic radiographs. Boxes illustrate the interquartile range, with the mean marked by a black square, median by a black line within the box, and minimum and maximum values as whiskers. Dots mark outliers, that is, data that significantly differ from the other observations. CAC type 4 (vessel-outlining CAC) showed the highest mean and median cIMT thickness.

The mean ± SD cIMT was significantly higher among individuals with any type of CAC on PR (0.83 ± 0.15 mm), compared to those without CAC on PR (0.77 ± 0.136 mm, *P* = .013). The most significant difference in mean ± SD cIMT was found between individuals with vessel-outlining CACs (type 4) on PR (0.88 ± 0.143 mm; median 0.90 mm, IQR 0.76-0.95) versus those with no CAC on PR (*P* = .011). Table 3 and Figure 4 present comparisons of cIMT between the groups with different types of CAC on PR.

**Table 3.** Carotid intima media thickness (cIMT) and plaque presence determined by carotid ultrasound, according to findings of carotid artery calcification (CAC) on panoramic radiographs.

		All included participants	No CAC	All CAC types	*P* value*	CAC type 1-3	*P* value*	Vessel-outlining CAC type 4	*P* value*
		*n *= 135	*n *= 88	*n *= 47		*n *= 31		*n *= 16	
cIMT	mean (SD)	0.79 (0.144)	0.77 (0.136)	0.83 (0.150)	**.013** ^a^	0.81 (0.151)	NA	0.88 (0.143)	**.011** ^b^
	median (IQR)	–	–	–	NA	0.82 (0.70-0.88)	.344^b^	0.90 (0.76-0.95)	NA
Participants with plaque	*n* (% presenting with both CAC and plaque)	76 (56.3)	38 (43.2)	38 (80.9)	**<.001** ^c^	24 (77.4)	**.001** ^c^	14 (87.5)	**.001** ^c^

* Compared with no CAC;

a Student’s t-test, two tailed;

b Kruskal-Wallis test with Bonferroni correction;

c Pearson’s chi-squared;

Abbreviation; NA = not applicable.

**Bolded *P*-values** are statistically significant (*P* <.05).

### Carotid ultrasound findings of plaque

The CUS examinations revealed plaque in 76 participants (56.3%). Those with versus without plaque on CUS did not significantly differ in mean age, sex, or months between PR and CUS examinations (Table 1). The prevalence of plaques detected by CUS significantly differed between participants presenting with any type of CAC on PR versus those without CAC on PR (*P* < .001). Among the 47 participants with any type of CAC on PR, 38 (80.9%) exhibited a plaque on CUS. In contrast, among the 88 participants without CAC on PR, only 38 (43%) exhibited a plaque upon CUS examination (Table 3).

**Figure 5.**
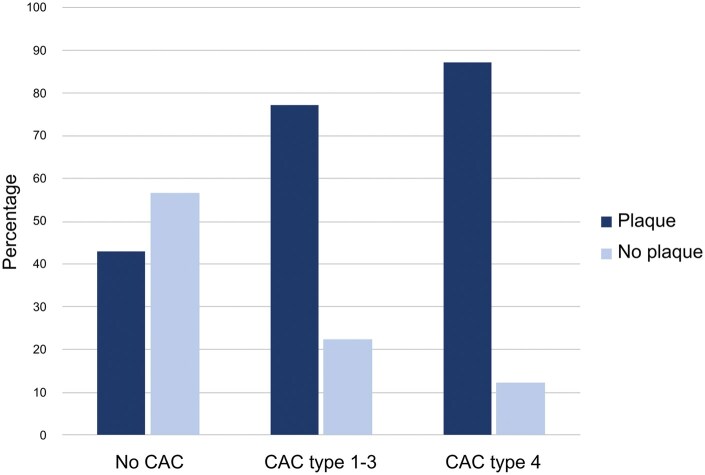
Percentages of participants with and without carotid artery calcifications (CACs) detected on panoramic radiographs, divided into types 1-3 or type 4, and in relation to plaque detected on carotid ultrasound. Significant differences were found within each group (*P* < .05).

When the participants were stratified by CAC type, the prevalence of plaques on CUS was higher among those with vessel-outlining CACs (type 4), compared to those with no CAC on PR (87.5% vs 43.2%, *P* < .001). Similarly, the prevalence of plaques on CUS was higher among participants with CAC type 1-3 on PR, compared to those without CAC on PR (77.4% vs 43.2%, *P* < .001) (Table 3, Figure 5).

The estimated sensitivity, specificity, PPV, NPV and accuracy för CAC in total, CAC type 1-3, and CAC type 4, when considering any CUS-detected plaque as a reference standard, are shown in Table 4.

**Table 4.** Diagnostic values of carotid artery calcification (CAC) in relation to plaque on carotid ultrasound as the reference standard.^a^

	All CAC types	CAC type 1-3	CAC type 4
	*n *= 47	*n *= 31	*n *= 16
Sensitivity (95% CI)	50% (38.4%-61.6%)	38.7% (26.9%-52.0%)	26.9% (16.0%-41.3%)
Specificity (95% CI)	84.7% (72.5%-92.4%)	87.7% (75.7%-94.5%)	96.2% (85.7%-99.3%)
Positive predictive value (95% CI)	80.9% (66.3%-90.4%)	77.4% (58.5%-89.7%)	87.5% (60.4%-97.8%)
Negative predictive value (95% CI)	56.8% (45.8%-67.2%)	56.8% (45.8%-67.2%)	56.8% (45.8%-67.2%)
Accuracy (95% CI)	65.2% (56.5%-73.2%)	62.2% (52.8-70.9%)	61.5% (51.5-70.9%)

a The group with no CAC was compared to the different groups distributed according to type of CAC: all CAC types, CAC type 1-3, and CAC type 4 (vessel-outlining CAC).

## Discussion

The main finding of the present study was that the detection of CACs on PRs was associated with the detection of plaque and increased cIMT on CUS, which are both indicators of atherosclerotic disease associate with elevated risk of future cardiovascular events, such as stroke and myocardial infarction. Moreover, we found that compared to other types of CACs, vessel-outlining CACs on PR were associated with more severe vascular disease according to the CUS findings. Our results suggest that dentists have an opportunity to identify patients at risk for cardiovascular-related morbidity and death, by assessing PRs for CACs, especially vessel-outlining CACs. These findings are in line with those of a recent prospective study that followed patients with vessel-outlining CAC for a mean of 7 years.^9^

The reported prevalence of CACs on PRs has widely varied between different study populations. The participants in our present study were 40, 50, and 60 years old at baseline (mean ± SD age at inclusion in the present sub-study population: 60.7 ± 5.7 years), each had at least one CVD risk factor, and exhibited an overall CAC prevalence of 34.8%. Similarly, a previous Swedish study included participants with a recent myocardial infarction (mean ± SD age: 62 ± 8 years), and found a CAC prevalence of 33.8%.^17^ In contrast, other studies of similarly aged populations, but not exclusively including individuals with CVD risk factors, have reported lower prevalences of CACs on PRs, ranging from 21.7% to 27.6%.^17^^,^^18^ Studies including wider age ranges, of 18-74 years and 2-92 years, have also reported lower CAC prevalence rates of 9.9% and 4%, respectively.^19^^,^^20^ The differing CAC prevalence between studies may be partly explained by the differences in exclusion criteria. Several studies, including ours, have excluded PRs that did not include the area of the carotid arteries,^10^^,^^17^^,^^18^ whereas other studies have categorized these as exhibiting no findings of CAC.^19^^,^^20^ Such exclusions contribute to the higher CAC prevalence in our study.

The prevalence of CACs on PRs increases with age, and this was conclusively demonstrated with our findings. The mean age significantly differed between participants presenting with CACs on PRs (62.7 years) versus those with no CAC on PR (59.6 years). Furthermore, participants with vessel-outlining CACs had the highest mean age (64.9 years; median 64.5 years, IQR 63-68). A previous study that was similar to our present investigation, and that included both participants with and without CACs, also reported correlations between CVD and CACs on PRs.^21^ However, that study included fewer participants (*n *= 105) and did not specifically include individuals with cardiovascular risk factors. Only a limited number of previous studies have examined the prevalence of specific types of CACs on PRs. In the present population, 8.2% of the neck sides had vessel-outlining CACs, which is slightly higher than in the 2 previous studies, which reported prevalence rates of between 5.2% and 5.6%.^6^^,^^10^^,^^17^ However, these studies included a wider age range, and did not exclusively include people with CVD risk factors, as in the present study.

When considering CUS examination as a reference standard for carotid plaque detection, PR-detected CACs of any type exhibited a specificity of 84.7%, and vessel-outlining CACs had an even higher specificity of 96.2%, and PPV of 87.9%. This means that detection of a type 4 CAC is associated with a higher probability that the patient has a carotid plaque. In contrast, CACs of any type showed a lower sensitivity (50%); and vessel-outlining CACs showed a sensitivity of only 26.9%. This means that the lack of CAC on a PR cannot be used to exclude that a patient may have carotid plaques and atherosclerosis.

Multiple factors may contribute to this lower sensitivity. First, atherosclerosis may develop over a long period of time, and PRs can only depict plaques with calcifications, which are less likely to be present during early stages of atherosclerosis.^5^ Typically, the complexity of calcification increases with a greater degree of luminal narrowing,^5^ although advanced carotid plaques are not always calcified. Therefore, when diagnosing carotid plaque, CACs on PRs have a lower sensitivity than CUS, because PR cannot detect soft tissue plaques without calcifications.^22^ Furthermore, the area of the carotid arteries may not always be visible on a PR due to anatomical variations, the area of the neck depicted on the PR, possible positioning errors, and the type of imaging equipment. The high specificity and PPV, and lower sensitivity and NPV, indicate that PR findings should not be used for screening carotid plaques. However, it may be beneficial for dentists to assess incidental findings of CACs on PRs performed for odontological reasons, because these findings correlate with CVD risk, especially when a vessel-outlining CAC is detected.

A few participants (*n *= 9) exhibited CAC on PR, but no plaques on CUS. One possible explanation is that in the VIPVIZA study, CUS was used to examine the carotid arteries for plaques in the area of the carotid bifurcation, and some CACs might have been situated below or above this area, and were thus not registered as plaque but still visible on PR. Another possible explanation is that the calcification could represents a calcified structure outside the artery.

We found that the mean cIMT significantly differed between participants with and without CACs. Few studies have examined the correlation between cIMT and CACs on PRs. One investigation included 4050 participants, with a CAC prevalence of 6.2% and a mean age similar to in our present study, and reported no significant differences in cIMT related to CACs.^16^ This may be explained by the fact that our study included only participants having at least one CVD risk factor, while their study included participants both with and without CVD risk factors.

One limitation of the present study is the small sample size. Among all 2570 participants who provided consent, only 168 had a PR taken within 18 months of a CUS examination, as revealed by a retrospective search of participants’ dental records. An additional 33 participants were excluded due to insufficient image quality for CAC detection. Moreover, since this was a retrospective study, all PRs had been performed for odontological indications; therefore, the area of the carotid arteries was not of particular interest and was not always depicted. It is also important to consider the risk of selection bias regarding the available PRs. A PR is often performed for patients requiring more advanced dental treatment. Another limitation is the varying time interval between the PR and CUS examinations (mean ± SD 8.71 ± 5.29 months). The participants underwent CUS examinations on 3 separate occasions during the VIPVIZA study, and this study included participants who underwent a PR a maximum of 18 months from any CUS examination. However, performing the PR and CUS examinations 18 months apart may not be optimal for comparisons. Another limitation is that the intervention in the VIPVIZA study included a pictorial presentation of atherosclerosis and recommendations on a healthier lifestyle, which was given to 48.9% of the participants at baseline. However, in this cross-sectional study, we compared CACs on PRs and CUS findings, and it is unlikely that the intervention affected the calcified parts in the plaque or the results. Although preventive treatment and recommendations might stop progression of the carotid plaque and CVD, the calcifications in the plaque and vessel walls will often remain.

Notably, all CAC assessments in this study were performed by 2 experienced researchers and specialists in oral and maxillofacial radiology. On the other hand, most PR examinations are performed by general dental practitioners. Therefore, the applicability of the present findings could be considered as a limitation, if dental practitioners cannot perform the assessment with the same accuracy. On PRs, CACs are usually located posteroinferior to the angle of the mandible, adjacent to the third or fourth cervical vertebra, but could be mistaken for other calcified structures in the area, such as a calcified triticeous cartilage or the superior cornu of the thyroid cartilage.^23^ However, a previous study reported that general dental practitioners who underwent a short 2-hour training programme about detecting CACs on PRs, and differentiating such calcifications from other calcified structures in the neck area, exhibited significantly improved diagnostic accuracy, with a sustained result over time.^24^ Those findings indicate that general dental practitioners can conduct this type of assessment with high specificity, which is important to avoid overloading the healthcare system with false-negatives. Although the sensitivity was lower, it would still be beneficial for the identified patients to seek medical attention if not already receiving CVD prevention. Importantly, vessel-outlining CACs may be easier for dentists to detect compared to other types of CACs.

## Conclusion

CACs of any type detected on PRs are associated with CUS findings of plaque and increased cIMT, which are both independent markers for atherosclerosis and CVD risk. In particular, the vessel-outlining CAC type indicates more severe vascular disease. By detecting these calcifications, especially vessel-outlining CACs, dentists could contribute to the early identification of patients with asymptomatic CVD, and recommend that they seek medical attention for preventive treatment.
